# Supplementary material for: Structural insights into IL-11-mediated signalling and human IL6ST variant-associated immunodeficiency
Source: Nat Commun. 2024 Mar 7;15:2071. doi: 10.1038/s41467-024-46235-6 (PMC10920896; doi:10.1038/s41467-024-46235-6)
Supplement: Supplementary file 1 — Supplementary Information [file 41467_2024_46235_MOESM1_ESM.pdf]

Structural insights into IL-11-mediated signalling and human *IL6ST* variant-associated immunodeficiency

Scott Gardner[1], Yibo Jin[1], Paul K. Fyfe[2], Tomas B. Voisin[1], Junel Sotolongo Bellón[3], Elizabeth Pohler[2], Jacob Piehler[3], Ignacio Moraga[2]*, Doryen Bubeck[1]*

[1] Department of Life Sciences, Sir Ernst Chain Building, Imperial College London, London, SW7 2AZ, UK.

[2] Division of Cell Signalling and Immunology, School of Life Sciences, University of Dundee, Dundee, United Kingdom.

[3] Department of Biology/Chemistry and Centre for Cellular Nanoanalytics, Osnabrück University, Osnabrück, Germany.

These authors contributed equally: Scott Gardner, Yibo Jin, Paul K. Fyfe

These authors jointly supervised this work: Ignacio Moraga, Doryen Bubeck

*Corresponding authors E-mail: d.bubeck@imperial.ac.uk (D.B.); imoragagonzalez@dundee.ac.uk (I.M.).

Supplementary information

Supplementary Figs 1-8

Supplementary Table 1

Supplementary Table 2

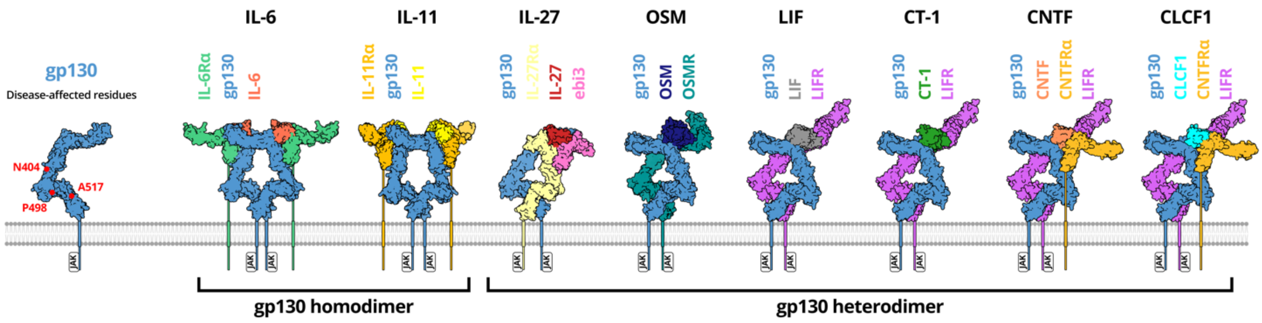

Supplementary Figure 1. Schematic of gp130 signalling complexes. Complexes that signal via dimerization of gp130 intracellular domains (IL-6 and IL-11) are grouped (gp130 homodimer). Those that signal through the coordination gp130 and co-receptor intracellular domains are indicated (gp130 heterodimer). Locations of mutations N404Y, P498L and A517P associated with hyper-IgE syndrome are highlighted on a gp130 monomer (red spheres).

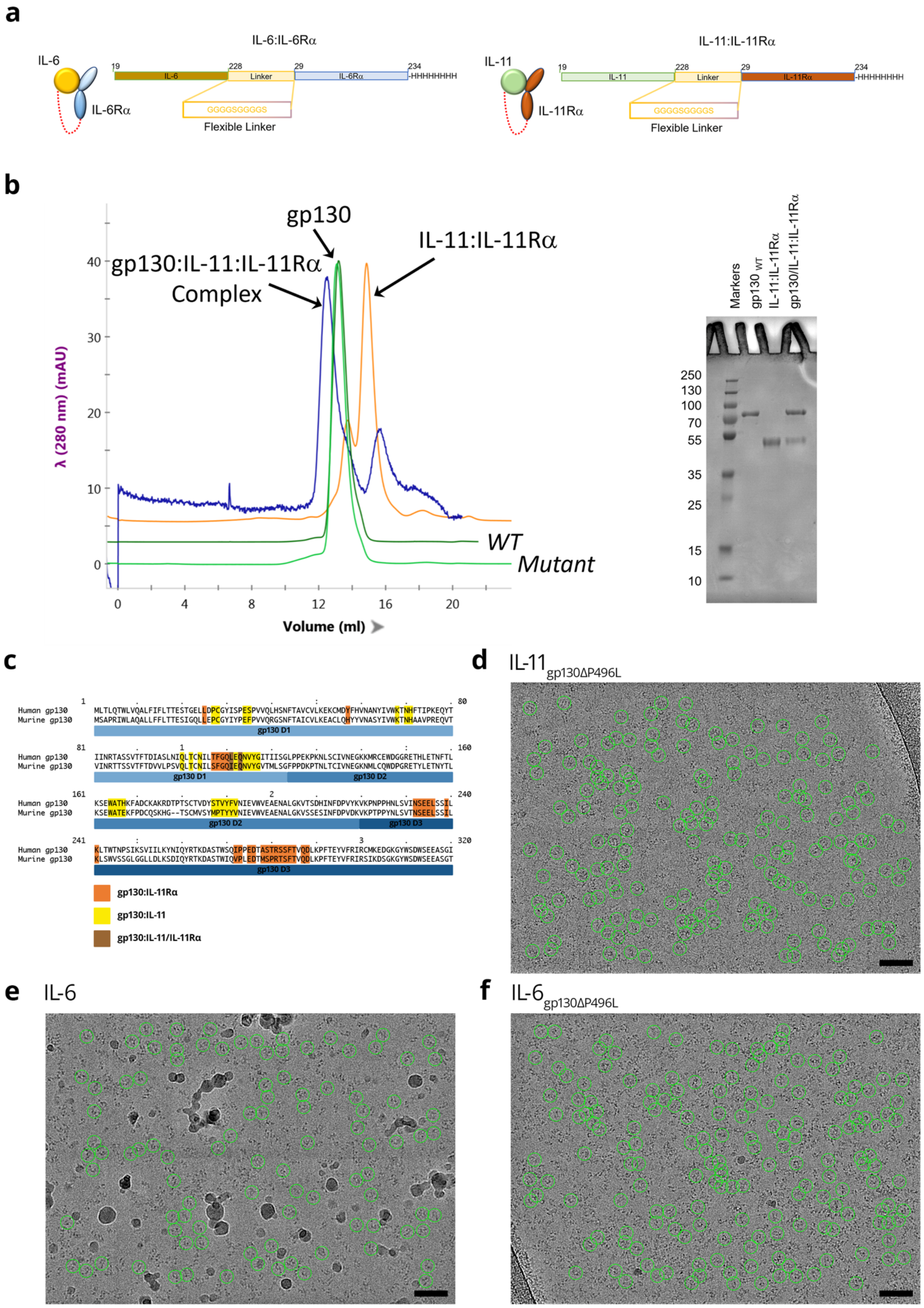
a
IL-6:IL-6Rα
IL-6
IL-6Rα
19
228
29
234
IL-6
Linker
IL-6Rα
-HHHHHHHH
GGGGSGGGGS
Flexible Linker
IL-11:IL-11Rα
IL-11
IL-11Rα
IL-11
Linker
IL-11Rα
-HHHHHHHH
GGGGSGGGGS
Flexible Linker
b
gp130
gp130:IL-11:IL-11Rα
Complex
IL-11:IL-11Rα
WT
Mutant
λ (280 nm) (mAU)
Volume (ml)
Markers
gp130 WT
IL-11:IL-11Rα
gp130/IL-11:IL-11Rα
250
130
100
70
55
35
25
15
10
c
Human gp130
Murine gp130
gp130 D1
gp130 D2
gp130 D3
gp130:IL-11Rα
gp130:IL-11
gp130:IL-11/IL-11Rα
d
IL-11gp130ΔP496L
e
IL-6
f
IL-6gp130ΔP496L

Supplementary Figure 2. Assembly and characterization of the IL-11 and IL-6 receptor complexes. (a) Schematic representation of the single chain IL-6 and IL-11 heterodimers. (b) Chromatogram overlay from size exclusion chromatography of the different IL-11 complex components. Coomassie-stained SDS-PAGE analysis under reducing conditions of the individual components and the IL-11-receptor complex used for cryoEM studies. (c) Sequence alignment of *murine* and *human* gp130; domains 1-3 shown in shades of blue bars below. Interface residues of the IL-11 receptor recognition complex are highlighted: gp130:IL-11Rα (red), gp130:IL-11 (yellow), and gp130:IL-11:IL-11Rα (brown). Representative cryoEM micrographs randomly selected of the IL-11 receptor recognition complex with gp130$_{P496L}$ (1 of 10,340) (d), IL-6 receptor recognition complex with wildtype gp130 (1 of 31,777) (e), and IL-6 receptor recognition complex with gp130$_{P496L}$ (1 of 10,795) (f). Scale bar 50 nm, particles included in the final reconstruction are circled.

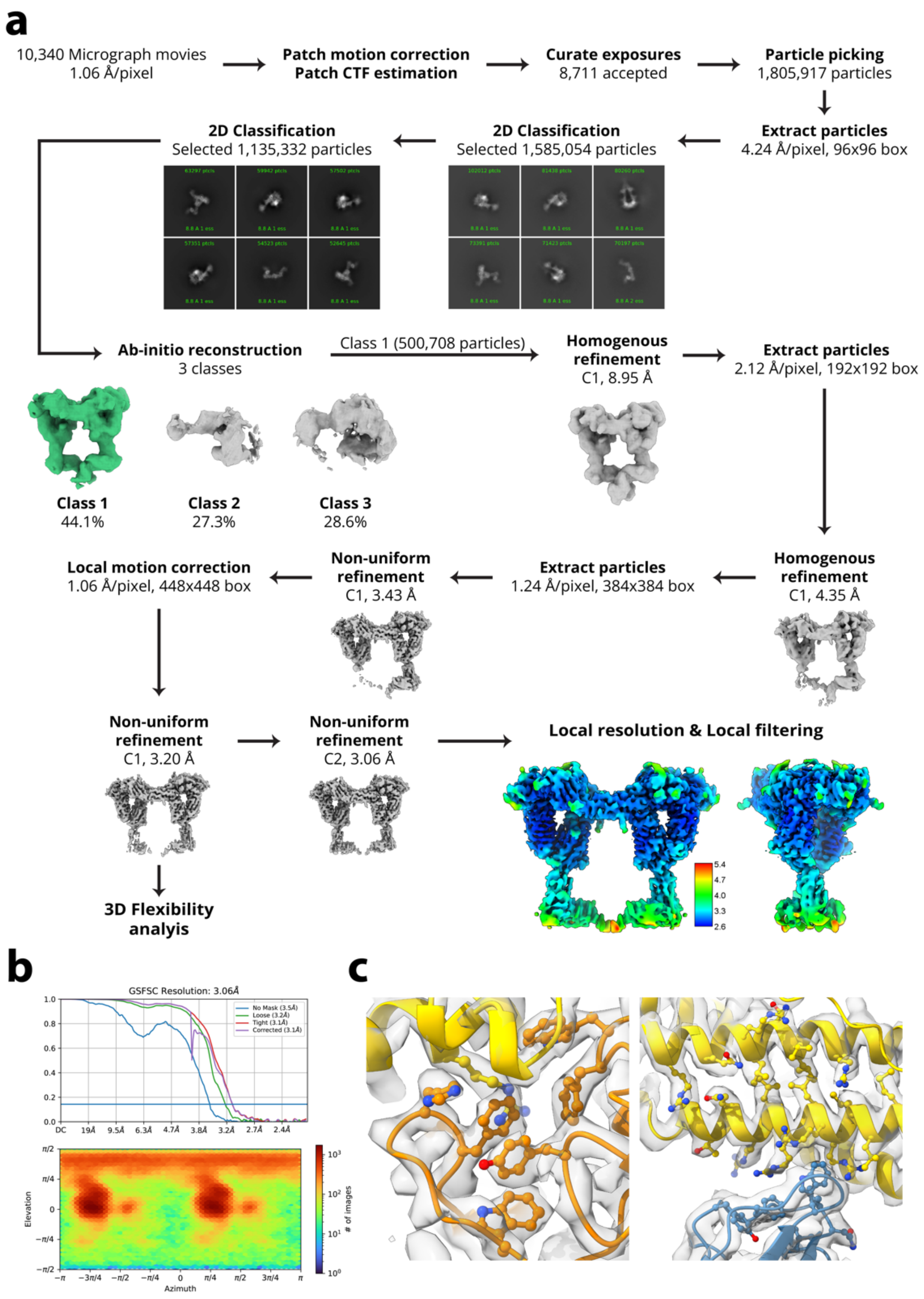

Supplementary Figure 3. Supplementary cryoEM data for the IL-11 receptor complex comprised of murine $gp130_{P496L}$. Schematic of cryoEM workflow, including a subset of representative 2D class averages (box size, 407 Å) and the final reconstruction filtered according to the local resolution ranging from 2.5 Å (blue) to 5.4 Å (red) (a). Gold standard Fourier shell correlation (GSFSC) for the final reconstruction (b, top panel), and angular distribution plot (b, bottom panel). The resolution at the 0.143 cutoff is reported. Map-model overlays for interface residues showing clear density for IL-11 (yellow), IL-11Rα (orange) and $gp130_{P496L}$ (blue) sidechains (c).

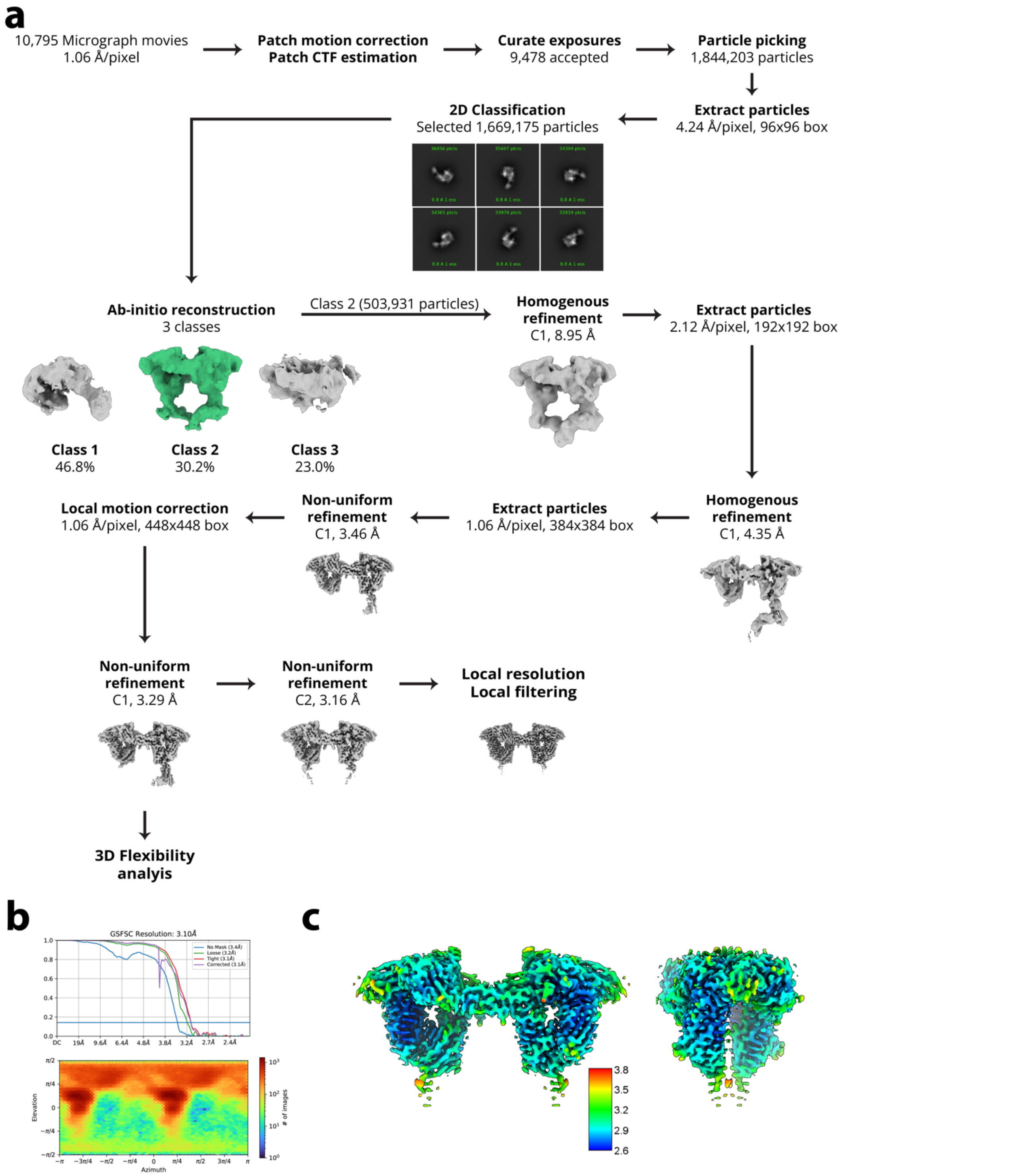

Supplementary Figure 4. Supplementary cryoEM data for the IL-6 receptor complex comprised of *murine* gp130$_{P496L}$. Schematic of cryoEM workflow, including a subset of representative 2D class averages (box size, 407 Å) (a). Gold standard Fourier shell correlation (GSFSC) for the final reconstruction (b, top panel), and angular distribution plot (b, bottom panel). The resolution at the 0.143 cutoff is reported. Final reconstruction filtered according to the local resolution ranging from 2.6 Å (blue) to 3.8 Å (red) (c).

a

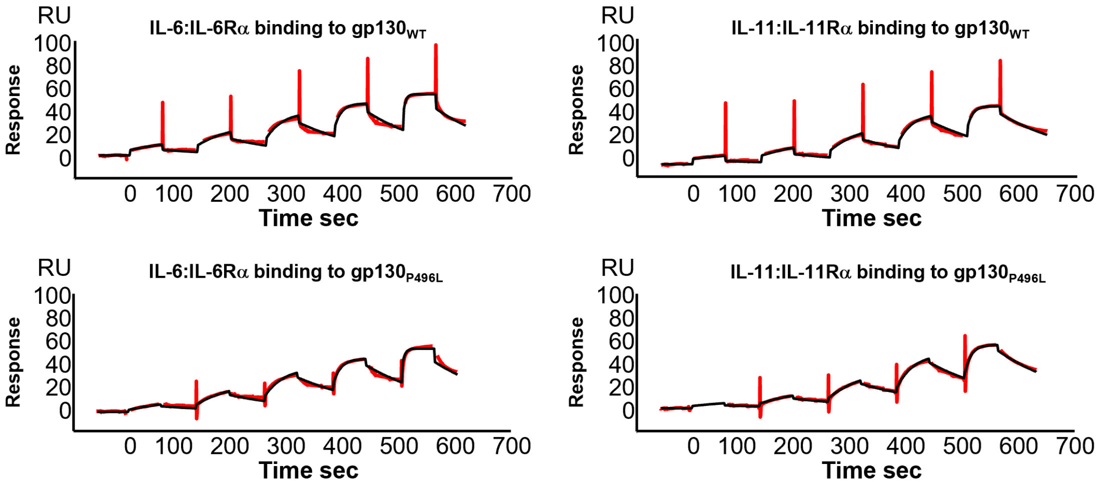

b

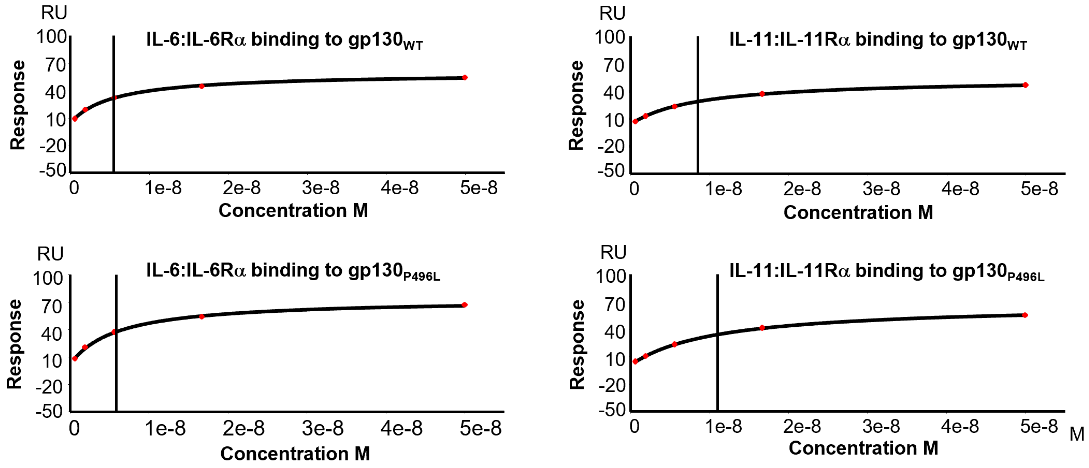

c

IL-6:IL-6Rα	k_{on} $M^{-1}s^{-1}$	k_{off} s^{-1}	$K_{DKinet.}$ nM	$K_{DEqui.}$ nM
gp130 $_{WT}$	2.6E+6<br>+/- 5.3E+4	9.7E-4<br>+/- 1.3E-4	0.37	5.5
gp130 $_{P498L}$	2.5E+6<br>+/- 4.1E+4	7.9E-4<br>+/- 9.9E-5	0.31	5.8

IL-11:IL-11Rα	k_{on} $M^{-1}s^{-1}$	k_{off} s^{-1}	$K_{DKinet.}$ nM	$K_{DEqui.}$ nM
gp130 $_{WT}$	1.5E+6<br>+/- 1.9E+4	0.0023<br>+/- 9.3E-5	1.45	8.5
gp130 $_{P498L}$	1.5E+6<br>+/- 1.3E+4	0.0027<br>+/- 8.0E-5	1.8	11

Supplementary Figure 5. Biochemical characterization of gp130 mutant. (a) SPR binding sensograms for the indicated ligands. *Murine* gp130 wt and mutant were immobilized on the chip surface by biotin-streptavidin interaction and IL-6:IL-6Rα or IL-11:IL-11Rα were flowed across the chip in solution. The binding constant values were estimated by fitting a pseudo first-order kinetic

model. (b) SPR equilibrium response fitting to a Langmuir model for the indicated conditions. (c) Kinetics and equilibrium binding constants for the gp130 and mutant are shown in table format.

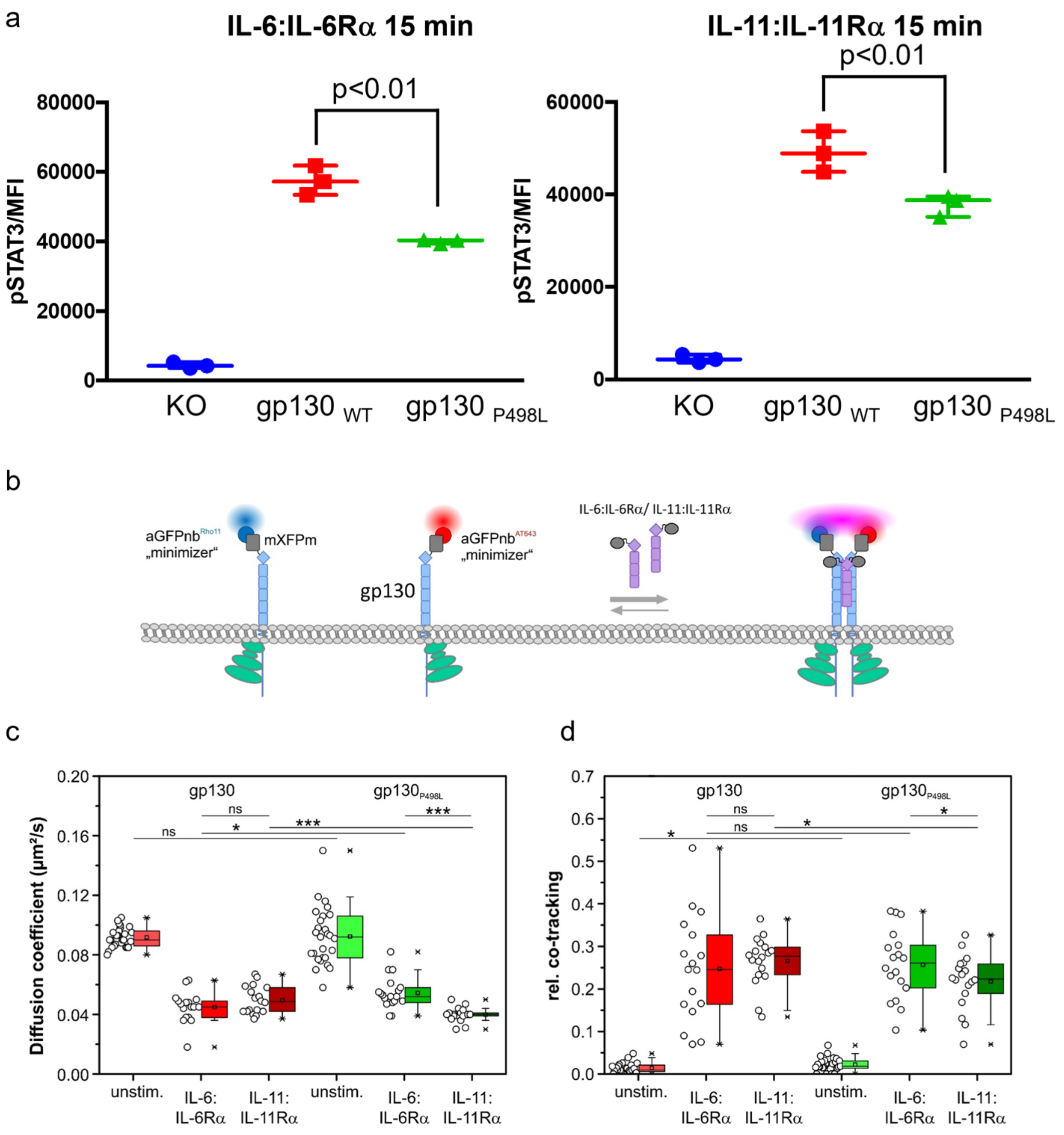

Supplementary Fig. 6 Functional characterization of gp130 mutant. (a) HeLa gp130 KO cells were transfected with gp130 wt or $gp130_{P498L}$ constructs and stimulated with 100 nM of IL-6:IL-6Rα or IL-11:IL-11Rα for 15 min. pSTAT1 and pSTAT3 levels were then measured by flow cytometry. Flow cytometry gating strategy is represented in the top panels. Data shown are the mean of three biological replicates with error bars depicting standard error of the mean. Significance was determined with a Two-tailed Student's T-test: $p<0.01$ (b) Cartoon of anti-GFP nanobody-based fluorescence labeling of cell-surface gp130 for single receptor localization and co-tracking. (c) Co-

diffusion of gp130 and $gp130_{P498L}$ in absence or presence of IL-6:IL-6Rα or IL-11:IL-11Rα. Each circle represents the relative dimerization of receptor subunits in an individual cell. (d) Diffusion coefficient of gp130 or $gp130_{P498L}$ subunits in unstimulated cells (unstim.) and for corresponding co-diffusion dimers after treatment with IL-6:IL-6Rα or IL-11:IL-11Rα. Each data point represents the mean diffusion constant in an individual cell. Asterisks in d and e represent the following p-values: ns – $p > 0.05$; * – $p \leq 0.05$; *** – $p \leq 0.001$. Data underlying panels a, c, and d are provided as Source Data.

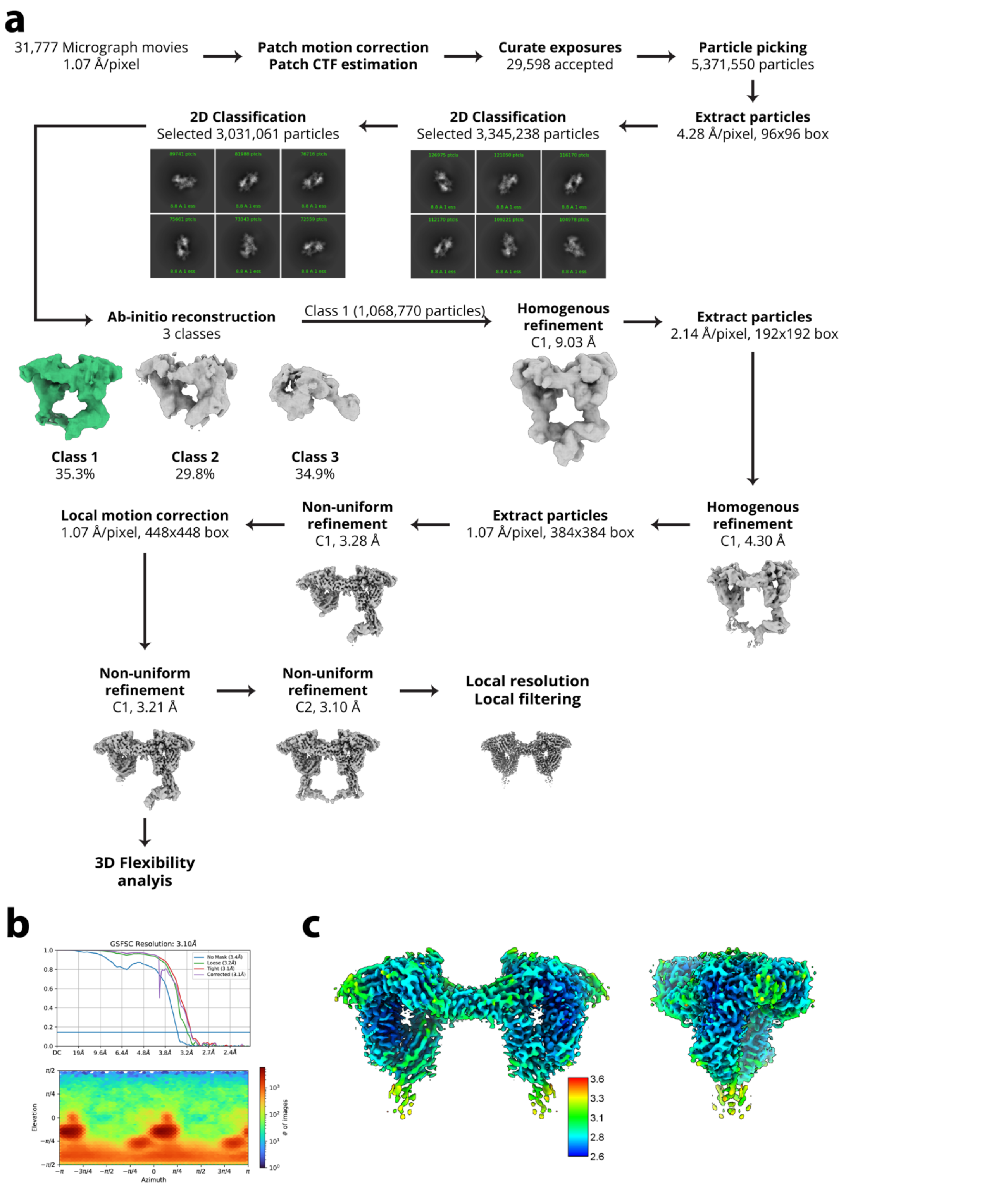

Supplementary Figure 7. Supplementary cryoEM data for the IL-6 receptor complex comprised of *murine* wildtype gp130. Schematic of cryoEM workflow, including a subset of representative 2D class averages (box size, 411 Å) (a). Gold standard Fourier shell correlation (GSFSC) for the final reconstruction (b, top panel), and angular distribution plot (b, bottom panel). The resolution at the 0.143 cutoff is reported. Final reconstruction filtered according to the local resolution ranging from 2.6 Å (blue) to 3.6 Å (red) (c).

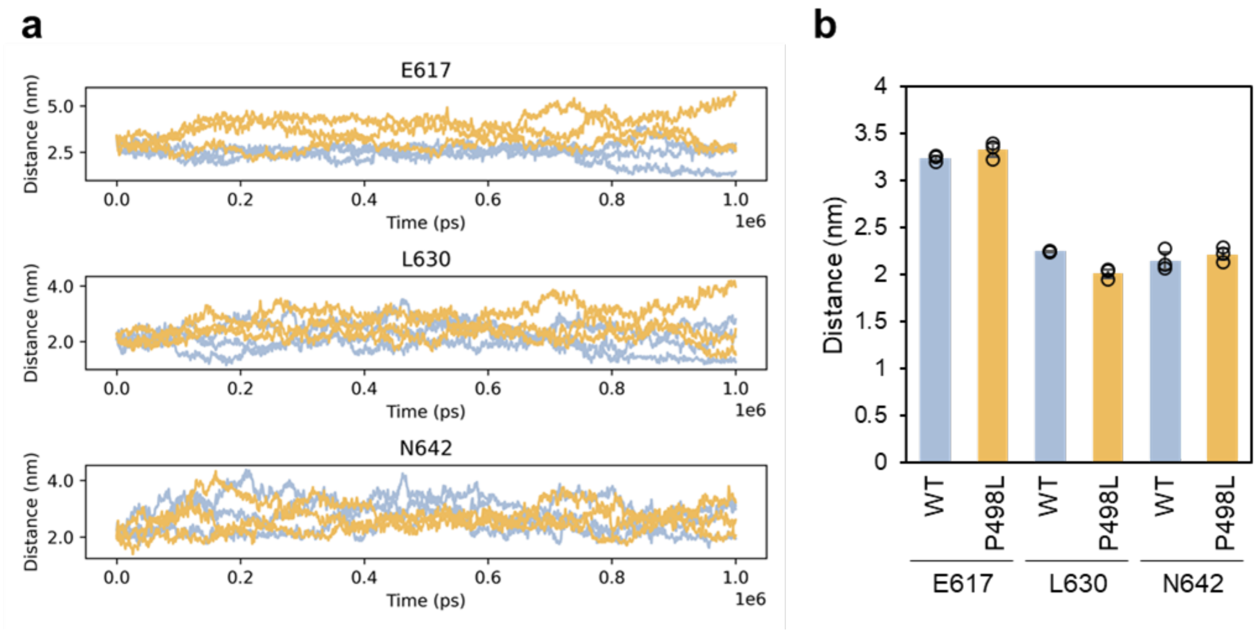

Supplementary Figure 8. Analysis of pairwise distances within the transmembrane domains of gp130. Time course of pairwise distances between the α carbon atoms of E617 (top), L630 (middle) and N642 (bottom) for the gp130 (blue) and gp130$_{P498L}$ (orange) within the transmembrane IL6 complex (a). Initial pairwise distances at the first step of the production runs indicating that the distances achieved in the simulations are independent of the initial configurations (b). The small differences observed are a result of prior equilibration runs.

Supplementary Table 1. CryoEM data collection and model validation statistics.

	IL-6 complex WT	IL-6 complex $gp130_{P496L}$	IL-11 complex $gp130_{P496L}$
EMDB	18742	18741	18743
PDB	8QY5	8QY4	8QY6
**Data collection**			
Microscope	Titan Krios	Titan Krios	Titan Krios
Voltage (keV)	300	300	300
Magnification	81,000	81,000	81,000
Electron exposure (e^-/A^2)	50.0	50.0	50.0
Defocus range (µm)	0.50 – 2.50	0.50 – 2.50	0.50 – 2.25
Detector	K3	K3	K3
Collection mode	Super resolution	Super resolution	Super resolution
Pixel size (Å)	1.07	1.06	1.06
# Micrograph movies	31,777	10,795	10,340
**Reconstruction**			
# Particles	1,042,507	491,673	487,147
Symmetry	C2	C2	C2
Map resolution (Å) (FSC threshold = 0.143)	3.10	3.16	3.06
Map resolution range (Å)	2.6 – 10.6	2.6 – 10.3	2.6 – 10.0
Map sharpening ($Å^2$)	-123.0	-143.3	-139.3
**Model refinement**			
Model resolution (FSC threshold = 0.5)	3.3	3.4	3.3
Number of residues			
Protein	1,884	1,884	1,890
Ligand	NAG: 22	NAG: 22	NAG: 22
B-factors ($Å^2$)			
Protein	67.52	25.97	28.95
Ligand	58.09	22.74	30.04
R.M.S. deviations			
Bond lengths (Å)	0.003	0.004	0.004
Bond angles (°)	0.736	0.757	0.846
**Validation**			
MolProbity score	1.56	1.64	1.56
Clash score	5.44	5.77	4.59
Rotamer outliers (%)	0.00	0.00	0.00
Cβ outliers (%)	0.00	0.00	0.00
Ramachandran plot			
Favoured (%)	96.15	95.29	95.3
Allowed (%)	3.85	4.71	4.7
Outliers (%)	0.00	0.00	0.00

Supplementary Table 2. System composition and box dimensions for the atomistic molecular dynamics simulations.

	**gp130**	**$gp130_{P498L}$**
Box dimensions (built by CHARMM-GUI)	25.04 × 25.04 × 25.61 nm	25.03 × 25.03 × 25.71 nm
**Composition**		
Protein		
Atoms	31630	31640
Chains	6	6
POPC		
Atoms	195104	195104
Molecules	1456	1456
Cholesterol		
Atoms	46176	46176
Molecules	624	624
Water (TIP3)		
Atoms	1252026	1256064
Molecules	417342	418688
Na^+	1166	1170
Cl^-	1162	1166
Total number of atoms	1527264	1531320
**Production runs**		
Length per replicate	1 μs	1 μs
Number of replicates	3	3
