# Supplementary material for: Structural insights into IL-11-mediated signalling and human IL6ST variant-associated immunodeficiency
Source: Nat Commun. 2024 Mar 7;15:2071. doi: 10.1038/s41467-024-46235-6 (PMC10920896; doi:10.1038/s41467-024-46235-6)
Supplement: Supplementary file 3 — Description of Additional Supplementary Files [file 41467_2024_46235_MOESM3_ESM.pdf]

File Name: Supplementary Movie 1

Description: 3D flexibility analysis of the IL-11 receptor complex comprised of murine $gp130_{P496L}$. Scatter plot showing the final distribution of learned particle coordinates (blue dots) across the dataset is shown in the inset. The orange and green lines indicate trajectories of the sampled motions. The movie shows an animation between maps corresponding to points (colored circles) along each of the trajectories. Approximate location of the equivalent disease affected residue on the murine $gp130_{P496L}$ is indicated by a black arrow.

File Name: Supplementary Movie 2

Description: 3D flexibility analysis of the IL-6 receptor complex comprised of murine wildtype gp130 (left panel) and murine $gp130_{P496L}$ (right panel). Scatter plot showing the final distribution of learned particle coordinates (blue dots) across the dataset is shown in the inset. The orange and green lines indicate trajectories of the sampled motions. The movie shows an animation between maps corresponding to points (colored circles) along each of the trajectories. Approximate location of the equivalent disease-affecting residue on the murine $gp130_{P496L}$ is indicated by a black arrow.
