# Supplementary material for: Structural insights into IL-11-mediated signalling and human IL6ST variant-associated immunodeficiency
Source: Nat Commun. 2024 Mar 7;15:2071. doi: 10.1038/s41467-024-46235-6 (PMC10920896; doi:10.1038/s41467-024-46235-6)
Supplement: Supplementary file 6 — Reporting Summary [file 41467_2024_46235_MOESM6_ESM.pdf]

nature portfolio

Corresponding author(s): Doryen Bubeck and Ignacio Moraga

Last updated by author(s): Feb 12, 2024

Reporting Summary

Nature Portfolio wishes to improve the reproducibility of the work that we publish. This form provides structure for consistency and transparency in reporting. For further information on Nature Portfolio policies, see our Editorial Policies and the Editorial Policy Checklist.

Statistics

For all statistical analyses, confirm that the following items are present in the figure legend, table legend, main text, or Methods section.

n/a	Confirmed	
☐	☒	The exact sample size (*n*) for each experimental group/condition, given as a discrete number and unit of measurement
☐	☒	A statement on whether measurements were taken from distinct samples or whether the same sample was measured repeatedly
☐	☒	The statistical test(s) used AND whether they are one- or two-sided<br>*Only common tests should be described solely by name; describe more complex techniques in the Methods section.*
☒	☐	A description of all covariates tested
☒	☐	A description of any assumptions or corrections, such as tests of normality and adjustment for multiple comparisons
☐	☒	A full description of the statistical parameters including central tendency (e.g. means) or other basic estimates (e.g. regression coefficient) AND variation (e.g. standard deviation) or associated estimates of uncertainty (e.g. confidence intervals)
☐	☒	For null hypothesis testing, the test statistic (e.g. *F*, *t*, *r*) with confidence intervals, effect sizes, degrees of freedom and *P* value noted<br>*Give P values as exact values whenever suitable.*
☒	☐	For Bayesian analysis, information on the choice of priors and Markov chain Monte Carlo settings
☒	☐	For hierarchical and complex designs, identification of the appropriate level for tests and full reporting of outcomes
☒	☐	Estimates of effect sizes (e.g. Cohen's *d*, Pearson's *r*), indicating how they were calculated

Our web collection on statistics for biologists contains articles on many of the points above.

Software and code

Policy information about availability of computer code

Data collection	CryoEM were collected with EPU software version 1.12.079 (Thermofisher Scientific). Molecular dynamics simulation systems were built using PPM 2, and CHARMM-GUI. Simulations were run using Gromacs 2021.3 with the CHARMM36m forcefield. SPR data was collected using Biacore T200 equipment. Phospho-flow data was collected using CytoFlex flow cytometer using Kaluza Analysis software version 1.3. Fluorescence microscopy data was collected using Andor iQ 2.4.4 software.
Data analysis	CryoEM data was analyzed with published softwares cryoSPARC v4.2.1, Topaz v0.2.5, ChimeraX v1.6.1, Coot v0.8.9, Phenix v1.21, PDBe PISA v1.52, and AlphaFold2. Protein sequences were aligned using the webserver ClustalW. Molecular dynamics simulations were analyzed using Gromacs 2021.3, ChimeraX v1.6.1, seaborn v0.11.1 and Matplotlib v3.3.4 in Python v3. SPR data was analyzed using T200 Evaluation Software v3.0. Phospho-flow data was analyzed using Prism software v7. Single molecule co-localization and co-tracking were analyzed using a custom-made MATLAB script SLIMfast 4C (https://doi.org/10.5281/zenodo.5712332).

For manuscripts utilizing custom algorithms or software that are central to the research but not yet described in published literature, software must be made available to editors and reviewers. We strongly encourage code deposition in a community repository (e.g. GitHub). See the Nature Portfolio guidelines for submitting code & software for further information.

Data

Policy information about availability of data

All manuscripts must include a data availability statement. This statement should provide the following information, where applicable:

- Accession codes, unique identifiers, or web links for publicly available datasets
- A description of any restrictions on data availability
- For clinical datasets or third party data, please ensure that the statement adheres to our policy

Data supporting the findings of this manuscript are available from the corresponding authors upon reasonable request. A reporting summary for this article is available as a Supplementary Information file. Source data are provided with this paper. Raw data generated in this study underlying Supplementary Fig. 6a, c and d are included as a source data file. CryoEM maps generated in this study have been deposited in the Electron Microscopy Data Bank under the accession codes EMD-18743 [https://www.ebi.ac.uk/pdbe/entry/emdb/EMD-18743] (IL-11:IL-11R⍰:gp130P496L); EMD-18741 [https://www.ebi.ac.uk/pdbe/entry/emdb/EMD-18741] (IL-6:IL-6R⍰:gp130P496L); and EMD-18742 [https://www.ebi.ac.uk/pdbe/entry/emdb/EMD-18742] (IL-6:IL-6R⍰:gp130). The structural models generated in this study have been deposited in the Protein Data bank under the accession codes 8QY6 [https://doi.org/10.2210/pdb8qy6/pdb] (IL-11:IL-11R ⍰:gp130P496L); 8QY4 [https://doi.org/10.2210/pdb8qy4/pdb] (IL-6:IL-6R⍰:gp130P496L); and 8QY5 [https://doi.org/10.2210/pdb8qy5/pdb] (IL-6:IL-6R⍰:gp130). Structural models used to initiate model building were accessed from the Protein Data Bank under the accession code 1P9M [https://doi.org/10.2210/pdb1p9m/pdb] and from the AlphaFold protein structure database entries Q00560 [https://alphafold.ebi.ac.uk/entry/Q00560]; A8K3F7 [https://alphafold.ebi.ac.uk/entry/A8K3F7]; and Q14626 [https://alphafold.ebi.ac.uk/entry/Q14626]. Structural model used to generate Fig. 2b and Fig. 2d were accessed from the Protein Data Bank under accession code: 6O4O [https://doi.org/10.2210/pdb6O4O/pdb]. Structural models used to generate Supplementary Fig.1 were accessed from the Protein Data Bank under accession codes: 7U7N [https://doi.org/10.2210/pdb7U7N/pdb]; 8D6A [https://doi.org/10.2210/pdb8D6A/pdb]; 8D74 [https://doi.org/10.2210/pdb8D74/pdb]; and 8D7R [https://doi.org/10.2210/pdb8D7R/pdb] and from the AlphaFold protein structure database entries P40189 [https://alphafold.ebi.ac.uk/entry/P40189]; Q6UWB1 [https://alphafold.ebi.ac.uk/entry/Q6UWB1]; Q99650 [https://alphafold.ebi.ac.uk/entry/Q99650]; P13725 [https://alphafold.ebi.ac.uk/entry/ P13725]; P42702 [https://alphafold.ebi.ac.uk/entry/P42702]; P26992 [https://alphafold.ebi.ac.uk/entry/ P26992]; and Q16619 [https://alphafold.ebi.ac.uk/entry/Q16619]. Molecular dynamics simulation data files produced in this study are uploaded to Zenodo database https://doi.org/10.5281/zenodo.10210284.

Research involving human participants, their data, or biological material

Policy information about studies with human participants or human data. See also policy information about sex, gender (identity/presentation), and sexual orientation and race, ethnicity and racism.

Reporting on sex and gender	N/A
Reporting on race, ethnicity, or other socially relevant groupings	N/A
Population characteristics	N/A
Recruitment	N/A
Ethics oversight	N/A

Note that full information on the approval of the study protocol must also be provided in the manuscript.

Field-specific reporting

Please select the one below that is the best fit for your research. If you are not sure, read the appropriate sections before making your selection.

☒ Life sciences ☐ Behavioural & social sciences ☐ Ecological, evolutionary & environmental sciences

For a reference copy of the document with all sections, see nature.com/documents/nr-reporting-summary-flat.pdf

Life sciences study design

All studies must disclose on these points even when the disclosure is negative.

Sample size	For structural studies of the gp130P496L:IL-11:IL-11Ra complex, 1,805,917 particles were picked from 10,340 electron micrograph movies. For structural studies of gp130wt:IL-6:IL-6Ra, 5,371,550 particles were picked from 31,777 micrograph movies. For structural studies of gp130P496L:IL-6:IL-6Ra 1,844,203 particles were picked from 10,795 micrograph movies. For fluorescence microscopy experiments, typically 15 individual cells were analyzed for each condition and statistical analysis is reported by unpaired student's t test. For single-molecule, phospho-Flow and SPR experiments, sample sizes were estimated based on extensive statistical analysis of data obtained in systematic assessment in previous studies: doi: 10.1016/j.crmeth.2022.100165; 10.15252/embr.202255450; 10.1126/scisignal.abc0653; 10.7554/eLife.49314 No sample size calculations were performed for the cryoEM data. The cryoEM data collected is sufficient for the resolution of the reported maps.
Data exclusions	For structural studies, electron micrograph movies with substantial drift and crystalline ice were excluded. This is a pre-established standard in the cryoEM community. Picked particles were excluded based on 2D and 3D classification. Excluded particles were those which belonged to classes that lacked high resolution structural features; this is a pre-established standard in the cryoEM community. For the single-molecule

	imaging, immobile particles classified as single molecules confined to a radius of 100nm for more than one second (30 frames) were removed. No data was excluded from SPR, phospho-flow, or MD simulations.
Replication	MD simulations were performed for three independent replicates. SPR and phospho-flow experiments were performed in three biological replicates. All attempts at replication were successful.
Randomization	Not relevant to this study, since samples were not allocated into experimental groups.
Blinding	Not relevant to this study, since there were no group allocations in this study.

Reporting for specific materials, systems and methods

We require information from authors about some types of materials, experimental systems and methods used in many studies. Here, indicate whether each material, system or method listed is relevant to your study. If you are not sure if a list item applies to your research, read the appropriate section before selecting a response.

Materials & experimental systems

n/a	Involved in the study
☐	☒ Antibodies
☐	☒ Eukaryotic cell lines
☐	☐ Palaeontology and archaeology
☐	☐ Animals and other organisms
☐	☐ Clinical data
☐	☐ Dual use research of concern
☐	☐ Plants

Methods

n/a	Involved in the study
☐	☐ ChIP-seq
☐	☒ Flow cytometry
☐	☐ MRI-based neuroimaging

Antibodies

Antibodies used	anti-pSTAT3Alexa488 - Biolegend #651006 clone 13A3-1 - dilution 1/50<br>Anti-GFP nanobody - In house expression - Used at 1.5 nM concentration
Validation	Validated by the manufacturer (https://www.biolegend.com/Files/Images/media_assets/pro_detail/more_data/13A3-1_STAT3_Phospho_MouseReactivityData_072716_updated.pdf)

Eukaryotic cell lines

Policy information about cell lines and Sex and Gender in Research

Cell line source(s)	HeLa gp130 KO cells were generated in the lab, and are derived from HeLa cells obtained from the German Collection of Microorganism and Cell Cultures GmbH (ACC 57).
Authentication	Not authenticated
Mycoplasma contamination	The cells were tested for Mycoplasma and found to be negative.
Commonly misidentified lines<br>(See ICLAC register)	None of the cell lines used in this study are commonly misidentified

Palaeontology and Archaeology

Specimen provenance	N/A
Specimen deposition	N/A
Dating methods	N/A

☐ Tick this box to confirm that the raw and calibrated dates are available in the paper or in Supplementary Information.

Ethics oversight	N/A

Note that full information on the approval of the study protocol must also be provided in the manuscript.

Animals and other research organisms

Policy information about studies involving animals; ARRIVE guidelines recommended for reporting animal research, and Sex and Gender in Research

Laboratory animals	N/A
Wild animals	N/A
Reporting on sex	N/A
Field-collected samples	N/A
Ethics oversight	N/A

Note that full information on the approval of the study protocol must also be provided in the manuscript.

Clinical data

Policy information about clinical studies

All manuscripts should comply with the ICMJE guidelines for publication of clinical research and a completed CONSORT checklist must be included with all submissions.

Clinical trial registration	N/A
Study protocol	N/A
Data collection	N/A
Outcomes	N/A

Dual use research of concern

Policy information about dual use research of concern

Hazards

Could the accidental, deliberate or reckless misuse of agents or technologies generated in the work, or the application of information presented in the manuscript, pose a threat to:

No	Yes	
☒	☐	Public health
☒	☐	National security
☒	☐	Crops and/or livestock
☒	☐	Ecosystems
☒	☐	Any other significant area

Experiments of concern

Does the work involve any of these experiments of concern:

No	Yes	
☒	☐	Demonstrate how to render a vaccine ineffective
☒	☐	Confer resistance to therapeutically useful antibiotics or antiviral agents
☒	☐	Enhance the virulence of a pathogen or render a nonpathogen virulent
☒	☐	Increase transmissibility of a pathogen
☒	☐	Alter the host range of a pathogen
☒	☐	Enable evasion of diagnostic/detection modalities
☒	☐	Enable the weaponization of a biological agent or toxin
☒	☐	Any other potentially harmful combination of experiments and agents

Plants

Seed stocks	N/A
Novel plant genotypes	N/A
Authentication	N/A

ChIP-seq

Data deposition

☐ Confirm that both raw and final processed data have been deposited in a public database such as GEO.

☐ Confirm that you have deposited or provided access to graph files (e.g. BED files) for the called peaks.

Data access links<br>*May remain private before publication.*	N/A
Files in database submission	N/A
Genome browser session<br>(e.g. UCSC)	N/A

Methodology

Replicates	N/A
Sequencing depth	N/A
Antibodies	N/A
Peak calling parameters	N/A
Data quality	N/A
Software	N/A

Flow Cytometry

Plots

Confirm that:

☒ The axis labels state the marker and fluorochrome used (e.g. CD4-FITC).

☒ The axis scales are clearly visible. Include numbers along axes only for bottom left plot of group (a 'group' is an analysis of identical markers).

☒ All plots are contour plots with outliers or pseudocolor plots.

☒ A numerical value for number of cells or percentage (with statistics) is provided.

Methodology

Sample preparation	Samples were fixed by quickly adding 15µl of PFA (16%) resulting in a final concentration of ~2% PFA solution and permeabilised for antibody staining in 500µl ice-cold Methanol.
Instrument	CytoFlex cytometer
Software	Kaluza Analysis software version 1.3
Cell population abundance	30,000 cells

Gating strategy | Cells were gated via FSC/SSC scatter in a first step and in GFP+ in a second step to identify gp130 expressing cells.

☒ Tick this box to confirm that a figure exemplifying the gating strategy is provided in the Supplementary Information.

Magnetic resonance imaging

Experimental design

Design type	N/A
Design specifications	N/A
Behavioral performance measures	N/A

Acquisition

Imaging type(s)	N/A
Field strength	N/A
Sequence & imaging parameters	N/A
Area of acquisition	N/A

Diabetes MRI ☐ Used ☐ Not used

Preprocessing

Preprocessing software	N/A
Normalization	N/A
Normalization template	N/A
Noise and artifact removal	N/A
Volume censoring	N/A

Statistical modeling & inference

Model type and settings	N/A
Effect(s) tested	N/A

Specify type of analysis: ☐ Whole brain ☐ ROI-based ☐ Both

Statistic type for inference (See Eklund et al. 2016)	N/A
Correction	N/A

Models & analysis

n/a	Involved in the study
☒	☐ Functional and/or effective connectivity
☒	☐ Graph analysis
☒	☐ Multivariate modeling or predictive analysis
